# Draft Genome Sequence of *Zobellella denitrificans* ZD1 (JCM 13380), a Salt-Tolerant Denitrifying Bacterium Capable of Producing Poly(3-Hydroxybutyrate)

**DOI:** 10.1128/genomeA.00948-17

**Published:** 2017-09-07

**Authors:** Yu-Wei Wu, Yiru Shao, Kamil Khanipov, Georgiy Golovko, Maria Pimenova, Yuriy Fofanov, Kung-Hui Chu

**Affiliations:** a Graduate Institute of Biomedical Informatics, College of Medical Science and Technology, Taipei Medical University, Taipei, Taiwan; b Zachary Department of Civil Engineering, Texas A&M University, College Station, Texas, USA; c Department of Pharmacology & Toxicology, The University of Texas Medical Branch at Galveston, Galveston, Texas, USA

## Abstract

*Zobellella denitrificans* ZD1, isolated from sediments of an estuarine mangrove ecosystem in Taiwan, exhibits growth-associated production of biopolymer poly(3-hydroxybutyrate) (PHB). This work reports the 4.05-Mbp draft genome sequence of *Z. denitrificans* ZD1, consisting of 217 contigs with a G+C content of 63.8% and 3,672 protein-coding sequences.

## GENOME ANNOUNCEMENT

*Zobellella*, a new genus, belongs to the gamma subgroup of *Proteobacteria*. *Zobellella denitrificans* ZD1 was isolated from sediment samples collected from the estuarine mangrove ecosystem of Chungkang, Miaoli County, Taiwan (1). Along with *Zobellella taiwanensis* ZT1, *Z. denitrificans* ZD1 was identified as one of the first two novel strains which are currently classified into the new *Zobellella* genus. *Z. denitrificans* ZD1 is characterized as a heterotrophic facultative anaerobic denitrifying bacterium. *Z. denitrificans* ZD1 is characterized as a Gram-negative straight rod, ranging from 1.6 to 2.6 mm long by 0.6 to 0.8 mm wide, with a single polar flagellum. The strain is also salt tolerant and capable of growth in the absence and presence of salt up to 12%, with optimal growth in 1 to 3% NaCl. The strain can also ferment different organics, including glucose, sucrose, and mannitol, and it can also use nitrate or nitrous oxide as an electron acceptor during denitrification. We recently observed a rapid growth of *Z. denitrificans* ZD1 with glycerol under aerobic conditions. The strain also exhibited growth-associated production of poly(3-hydroxybutyrate) (PHB), approximately 84% (wt/wt) of the dry cell weight (2), suggesting that this strain can be an ideal biocatalyst for PHB production.

The *Z. denitrificans* ZD1 strain was obtained from the Japan Collection of Microorganisms (JCM, Tsukuba, Japan). The genomic DNA was obtained from cells grown in R2A broth. The genomic DNA was processed into a sequencing library using a Nextera XT library preparation kit, according to the manufacturer guidelines. The sample was then barcoded using a Nextera XT index kit version 2. The draft genome of *Z. denitrificans* ZD1 was sequenced by 250-bp paired-end sequencing on an Illumina MiSeq sequencing system. After trimming adaptors and low-quality regions using bbduk (http://jgi.doe.gov/data-and-tools/bbtools/), the sequencing reads (two million pairs) were assembled *de novo* using SPAdes 3.10.1 (with the -careful option) (3). The final assembly of the genome produced 4,051,699 bp in 217 contigs, with an *N*_50_ value of 33,403 bp and a G+C content of 63.8%. The genome coverage is 101×. The assembled contigs were functionally annotated using the NCBI prokaryotic genome annotation pipeline (PGAP) annotation system (4). In total, 3,672 protein-coding genes along with 114 RNA genes were annotated. One clustered regularly interspaced short palindromic repeat (CRISPR) array spanning 7.1 kbp along with upstream CRISPR-associated (Cas) genes was also identified from the genome.

### Accession number(s).

The draft genome sequence of *Z. denitrificans* ZD1 has been deposited in DDBJ/EMBL/GenBank with the accession number NMUO00000000.
